# The lncRNA UCA1 promotes proliferation, migration, immune escape and inhibits apoptosis in gastric cancer by sponging anti-tumor miRNAs

**DOI:** 10.1186/s12943-019-1032-0

**Published:** 2019-07-04

**Authors:** Chao-Jie Wang, Chun-Chao Zhu, Jia Xu, Ming Wang, Wen-Yi Zhao, Qiang Liu, Gang Zhao, Zi-Zhen Zhang

**Affiliations:** Department of Gastrointestinal Surgery, Ren Ji Hospital, School of Medicine, Shanghai Jiao Tong University, No. 160 Pu Jian Road, Shanghai, 200127 China

**Keywords:** lncRNA, UCA1, Gastric cancer, miRNA, PDL1

## Abstract

**Background:**

UCA1 is a long non-coding RNA which was found overexpressed in various human cancers including gastric cancer (GC). It is identified that UCA1 promotes GC cells proliferation, migration and invasion, however, the role of UCA1 during the processes of immune escape is still not unclear.

**Methods:**

We collected 40 paired GC and non-tumor tissue samples. The level of UCA1 in GC and control tissue samples were determined by in situ hybridization and qRT-PCR. Cell viability was determined by MTT assay. GC cells’ migration capacities were examined by transwell assay. To understand the roles of UCA1 during immune escape, wildtype or UCA1 KO GC cells co-cultured with peripheral blood mononuclear cells or cytokine-induced killer cells in vitro. Mouse model was used to examine the function of UCA1 in vivo.

**Results:**

UCA1 promoted GC cells proliferation and migration, and inhibit apoptosis. UCA1 repressed miR-26a/b, miR-193a and miR-214 expression through direct interaction and then up-regulated the expression of PDL1. UCA1-KO GC cells could induce a higher IFNγ expression when co-cultured with peripheral blood mononuclear cells (PBMCs), and have a lower survival rate when co-cultured with cytokine-induced killer (CIK) cells in vitro. UCA1-KO GC cells formed smaller tumors, had higher miR-26a, −26b, −193a and − 214 level, reduced cell proliferation and increased apoptosis in xenograft mouse model.

**Conclusions:**

UCA1 overexpression protected PDL1 expression from the repression of miRNAs and contributed to the GC cells immune escape. UCA1 could serve as a potential novel therapeutic target for GC treatment.

**Electronic supplementary material:**

The online version of this article (10.1186/s12943-019-1032-0) contains supplementary material, which is available to authorized users.

## Introduction

As the fourth most common cancer worldwide, gastric cancer (GC) is still incurable and induces about 700,000 mortalities each year [1–4]. What is worse, non-resectable or metastatic GC is associated with poor prognosis, and systemic chemotherapeutic approaches provide minimal benefit. Thus, novel biomarkers for improving GC, early diagnosis, prognostic evaluation and tumor grading is urgently needed.

Owing to the development of continued advances in high throughput sequencing, scientists realized that the most part of human genome is transcribed to non-protein coding RNAs, which indicates that a large group of RNA regulators is dedicated to regulating a relatively small amount of effectors [5–7]. Among the newly discovered RNA elements, long non-coding RNAs (lncRNAs) have been identified to function as key regulators of diverse cellular processes, such as development, differentiation, and cell fate as well as disease pathogenesis [8, 9]. lncRNAs can serve as signal mediators, molecular decoys and scaffold or enhancers of transcription and what is intriguing is that a large group of lncRNAs function as competing endogenous RNA (ceRNA) that regulate gene expression through absorbing miRNAs [10].

Until now, different groups have screened the expression profile of lncRNAs in GC tumors and found several disordered lncRNAs relates to GC carcinogenesis such as PVT1, H19, LNC00152 and so on [11–13]. However, the underling mechanisms are still not well understood.

UCA1 (urothelial carcinoma associated 1) is a lncRNA that was first identified in human bladder carcinoma, whose expression is found to be up-regulated in many other cancers till now [14]. In gastric cancer, overexpressed UCA1 has been found in tumor tissues compared with paired non-cancerous samples, and the function of UCA1 have been confirmed to contribute to the cancer cell migration, invasion and drug resistance [15, 16].

In this study, we examined the level of UCA1 in different GC subtype tissue samples and unveiled the mechanism of how UCA1 modulates GC cells proliferation, migration and immune escape.

## Materials and methods

### Cell lines and human gastric cancer tissues

Human gastric cancer cell line AGS, SGC-7901, BGC-823, MGC-803 and SNU-1 were obtained from National Infrastructure of Cell Line Resource (Beijing, China). Primary stomach epithelial cells from two healthy individuals were purchased from Abace-biology (Beijing, China) (www.abace-biology.com/primary-cell.htm) All the cells were maintained in humidified incubator at 37 °C in a CO2 incubator in RPMI 1640 medium supplemented with 10% fetal bovine serum (FBS) and 1% penicillin-streptomycin.

A total of 40 primary gastric adenocarcinoma tumor tissues and corresponding adjacent non-tumorous gastric tissue samples were obtained between 2013 and 2015 at Ren Ji Hospital, Shanghai Jiao Tong University. A part of each tissue samples was subject to formalin fixation and paraffin-embedment. And another part of each tissue samples was stored in a refrigerator at − 80 °C. All the 40 paired tumor and non-tumor tissues were subjected to following study including immunoblotting, RNA extraction and qRT-PCR.

### Plasmid construction

Full length of 2314 UCA1 sequence was cloned into pCR3.1 vector to construct the UCA1 overexpression vector. For dual-luciferase assay, full length of UCA1 was cloned into pmirGLO vector, following firefly luciferase coding region. A 621 bp segment of PDL1 3’UTR was cloned into pmirGLO vector to form PDL1 3’UTR luciferase reporter vector.

For UCA1 knockdown construction, two guide RNAs targeting the promoter region of UCA1 were constructed into lenti-guide-puro vector (Addgene: 52963), named pUCA1-KD. For UCA1 knockout cell establishment, AGS or SGC-7901 cells were co-transfected with pUCA1-KD and plentiCas9-Blast (Addgene: 52962) followed by selection for more than 5 days with blasticidin (4μg/ml) and puromycin(2μg/ml) for 1 week.

### RNA real-time RT-qPCR

Quantitive RT-PCR analysis was used to determine the relative expression level of mRNAs and miRNAs. Total RNA was extracted from clinical samples and cells, using Trizol Reagent (Invitrogen, Carlsbad, CA, USA) according to the manufacturer’s instructions. The RNA samples were treated using TURBO DNA-free kit to remove the DNA contamination and then the DNA contamination was assessed by agarose gel. RevertAid First Strand cDNA Synthesis Kit (Thermo Scientific) was employed for the reverse-transcription, and the level of specific RNAs were determined using SYBR® Green real-time PCR kit (Invitrogen, Carlsbad, CA, USA) with GADPH as loading control. The level of β-actin was also quantified to confirm the relative expression of UCA1.

The expression level of miRNAs was detected by TaqMan miRNA RT-Real Time PCR. Single-stranded cDNA was synthesized by using TaqMan MicroRNA Reverse Transcription Kit (Applied Biosystems, Foster City, CA, USA) and then amplified by using TaqMan Universal PCR Master Mix (Applied Biosystems, Foster City, CA, USA) together with miRNA-specific TaqMan MGB probes (Applied Biosystems, Foster City, CA, USA). The U6 snRNA was used for loading control. The level of small nucleolar RNA, C/D box 58B (RNU58B) was also quantified to confirm the relative expression of miRNAs. Each sample in each group was measured in triplicate and the experiment was repeated at least three times for the detection of miRNAs.

### RNA in situ hybridization

Slides were deparaffinized and rehydrated through immersion in xylene and an ethanol gradient and then digested with proteinase K (20 μg/mL) for 20 min at 37 °C. Slides were firstly fixed in 4% paraformaldehyde for 5 min at room temperature, and then dehydrated by immersion in an ethanol gradient and air dried; slides were pre-hybridized using DIG Easy Hyb (Roche, Mannheim, Germany) at 50 °C for 1 h. The 10 pmol digoxin-labeled UCA1 DNA probe was denatured in hybridization buffer at 95 °C for 2 min and then chilled on ice. The UCA1 probe was diluted in 250 μL pre-warmed in hybridization buffer. Each sample was covered with 50–100 μL diluted probe and incubated in a humidified hybridization chamber at 50 °C overnight. Slides were washed twice in 50% formamide in 4 × SSC at 37 °C for 30 min, and then washed three times in 2 × SSC at 37 °C for 15 min. After twice washing with maleic acid buffer containing Tween-20 (MABT), slides were blocked using blocking buffer (Roche, Mannheim, Germany) at room temperature for 30 min, the blocking buffer was drained off, and the samples were incubated with 1:250 diluted Anti-Digoxigenin-AP Fab fragments (Roche, Mannheim, Germany) at 37 °C for 1 h. After washing twice in MABT and once in detection solution (0.1 M Tris-HCl, 0.1 M NaCl, pH 9.5), the slides were stained with freshly diluted NBT/BCIP detection solution (Roche, Mannheim, Germany) and incubated at 37 °C for 30 min. Slides were washed in PBS twice, air dried for 30 min, and then mounted with Eukitt quick-hardening mounting medium (Sigma Aldrich, St. Louis, MO, USA). Images were obtained using a microscopy (Leica DM2000 LED) and a digital camera (Leica DMC 2900). Three different random images were captured for each sample at 400× magnification and the relative density of UCA1 signal was quantified by Image J. The results were analyzed using paired t-test and *p* < 0.05 was statistically significant.

### Fluorescence in situ hybridization (FISH)

AGS cells were rinsed by PBS and then fixed in 4% formaldehyde for 10 min at RT. Further, the cells were permeabilized in PBS containing 0.5% Triton X-100 and 5 mM VRC(New England Biolabs Inc., USA) on ice for 10 min, then washed with PBS. After rinsed once in 2 × SSC, hybridization was carried out using FAM labelled UCA1 DNA probe at 37 °C for 12 h. After three times washing by 2 × SSC, one of the candidate miRNAs was detected by incubation with Cy5 labelled miRNA specific probe at 37 °C for 12 h. After nucleus staining by DAPI, images were captured by confocal microscopy (Leica TCS SP8) at 630× magnification.

### RNA antisense purification (RAP)

Cell extracts were incubated with biotin labelled UCA1 probe at 4 °C for 2 h, with biotin labelled sequence scrambled DNA oligo as control. Hybridized material was captured with magnetic streptavidin beads (Thermo Fisher Scientific). Beads were subjected to RNA extraction followed by RT-qPCR to quantify miRNAs levels.

### Cell apoptosis assay

The number of apoptotic cells were evaluated by assaying for annexin V. Cells were stained with FITC labeled Annexin V antibody and PI (BioLegend), and then cells were analyzed using flow cytometry. Results were analyzed using Flowjo software (Ashland).

### Cell proliferation assay

AGS and SGC-7901 cells were seeded in 96-well plates at density of 5 × 103 cells per well in RPMI 1640 culture, and allowed to attach overnight. pCR3.1-UCA1 was transfected into cells for 48 h, with empty vector as control. Twenty microliters MTT (5 mg/ml) (Sigma, St. Louis, MO, USA) were added into each well and the cells were incubated for further 4 h. The absorbance was recorded at A570nm with a 96-well plate reader after the DMSO addition.

### In vitro migration assay

Indicated cells were deprived of serum overnight, treated with mitomycin-C, seed into the upper chamber at density of 1 × 105/well (8 μm pore size; BD Bioscience). The chemoattractant in the lower chamber was filled with medium supplemented with 10% FBS. After 12 h, the lower chambers cells were fixed with 4% paraformaldehyde and then stained with crystal violet. The migrated cells were counted in 5 randomly different fields with an inverted microscope. The experiments were performed in triplicate wells and each experiment was performed in triplicate.

### Generation mouse xenograft model

The experiments including animals were approved by Ren Ji Hospital Animal Care and Use Committee. Female severe combined immunodeficient (SCID) beige mice were purchased from Charles River Laboratories at age 8 weeks with a weight between 20 g and 25 g. A total of 5 × 10^6^SGC-7901 cells in 100 μL PBS together with an equal volume of Matrigel basement membrane matrix were injected subcutaneously into the shoulder to establish a human GC cell xenograft model.

### Immunoblotting

Protein samples were boiled in SDS/β-mercaptoethanol sample buffer, and 20 μg of each sample was loaded into each lane of 4–12% polyacrylamide gels. After separation by electrophoresis, the proteins in the gels were transferred onto PVDF membranes (Amersham Pharmacia Biotech, St. Albans, Herts, UK). The membrane was incubated with rabbit anti-CDK6 polyclonal antibody (ab151247, Abcam, Cambridge, MA, USA) or rabbit anti-EZH2 monoclonal antibody (ab191080, Abcam, Cambridge, MA, USA) or rabbit anti-PDL1 polyclonal antibody (ab205921, Abcam, Cambridge, MA, USA) or rabbit anti-TAK1 polyclonal antibody (ab109526, Abcam, Cambridge, MA, USA) or rabbit anti-KRAS polyclonal antibody (ab180772, Abcam, Cambridge, MA, USA), or rabbit anti-FGF9 polyclonal antibody (ab71395, Abcam, Cambridge, MA, USA) or mouse anti-β-actin monoclonal antibody (Santa Cruz Biotechnology Inc., Santa Cruz, CA, USA) over night at 4 °C. The specific protein-antibody complex was detected by using horseradish peroxidase conjugated goat anti-rabbit or rabbit anti-mouse IgG. Detection by the chemiluminescence reaction was carried using the ECL kit (Pierce, Appleton, WI, USA). The β-actin signal was used as a loading control.

### Immunohistochemistry

Paraffin-embedded sections were firstly deparaffinized and then incubated with rabbit polyclonal anti-Ki-67 (ab15580, Abcam, Cambridge, MA, USA) or rabbit polyclonal anti-PDL1 (ab205921, Abcam, Cambridge, MA, USA) primary antibody at 4 °C overnight. After three times wash by TBST, the sections were then incubation with HRP conjugated goat anti-mouse or goat anti-rabbit secondary antibody. The sections were washed by TBST for three times and the signal was detected using DAB Substrate kit following the manufacture’s instruction. Images were obtained using a microscopy (Leica).

### CIK cells preparation

The peripheral blood was obtained from patients using heparin as the anticoagulant. The peripheral blood mononuclear cells (PBMCs) were isolated by Ficoll-Conray density gradient centrifugation, as described previously [17].The PBMCs (2.0 × 106/ml) were plated onto 6-well dishes and cultured with RPMI 1640 in the presence of human IFNγ (1.0 × 106 U/L); recombinant human interleukin 2 (5.0 × 105 U/L); 10% inactivated human serum; 25 mM HEPES; and 2 mM l-glutamine. After 24 h, monoclonal antibody against CD3 (100 μg/L, Antibody Diagnostic Inc., New York, NY, USA) and interleukin-1 alpha (1.0 × 105 U/L) were added. 2 days after incubation, the cells were cultured in medium without IFNγ and the medium was replaced every 3 days.

### Cytotoxicity activity analysis

For the cytotoxicity analysis, UCA1-KO and control GC cells were used as the target cells, while CIK cells were used as the effector cells. The effector and target cells were co-cultured in triplicate at ratios of 10:1, 20:1 and 40:1 with the wells containing just effector cells or target cells as controls. The cells were maintained in a humidified incubator at 37 °C in 5% CO2 for 24 h. 100 μl medium from the co-cultured cells was mixed with 20 μl CCK-8 solutions, and then incubated for another 4 h. The optical density (OD) was recorded at 450 nm for each well by a microplate reader. The survival (%) = (effector target cell mixture-effector group)/target cell*100%.

### Statistical analysis

Two-tailed Student’s t test was used to calculate statistical significance between two comparator groups. The differences of lncRNA expressions between paired tissue sample was evaluated with Wilcox matched pairs signed ranks test. The correlation analysis was analyzed by χ2-analysis. ALL the data were analyzed by using SPSS Statistical Package version 16. The survival times of different groups of patients were analyzed using the Kaplan-Meier method. A *p* value < 0.05 was considered as statistically significant.

## Results

### Increased UCA1 was found in patients with GC and related with poor prognosis in patients with intestinal GC

**Fig. 1:**
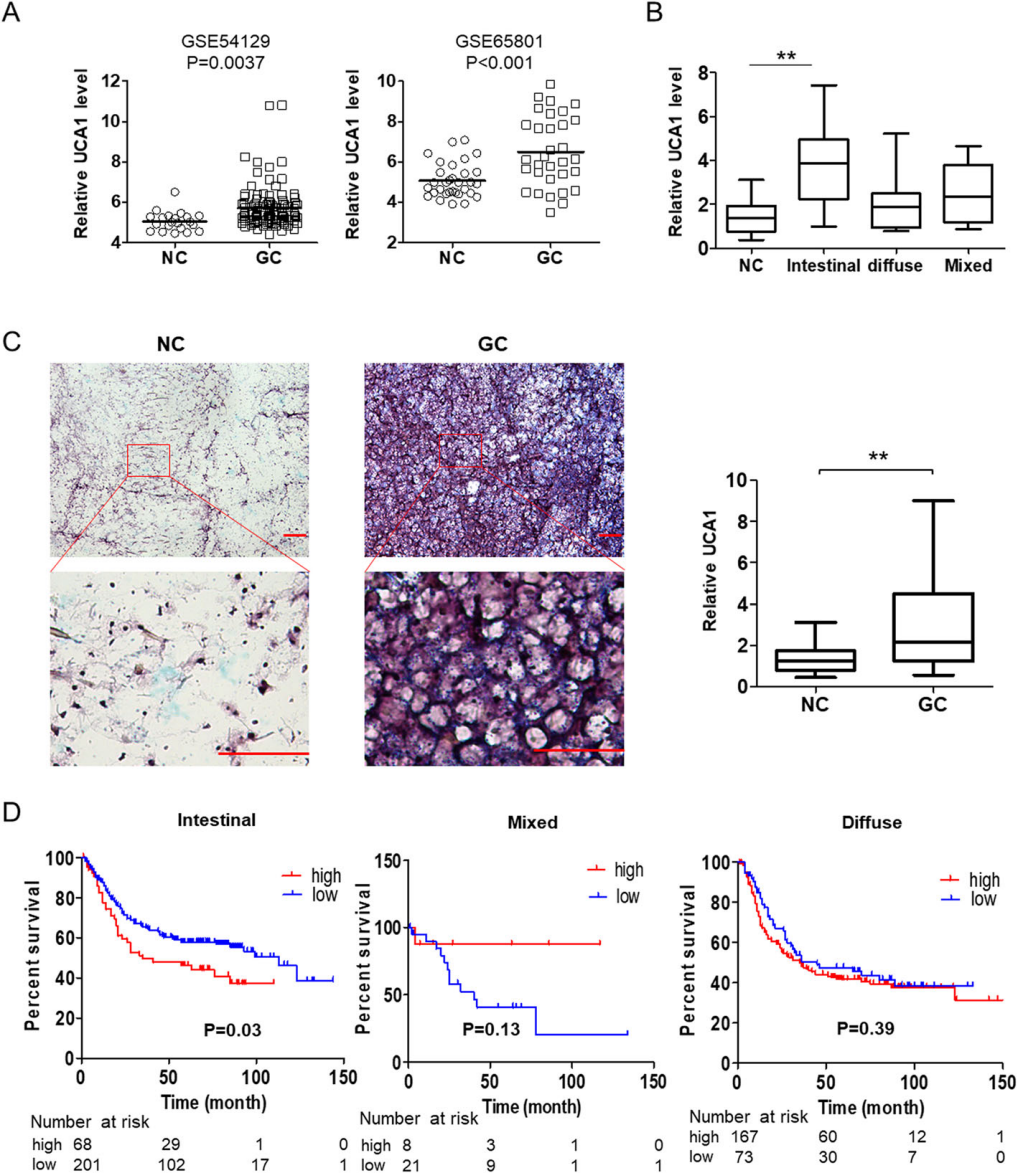
dysregulated UCA1 was found in GC tissues and relates to worse prognosis. **a** The UCA1 level from two microarray datasets (GSE54129and GSE65801) was analyzed by student’s t-test. *P* < 0.05 was considered statistically significant. **b** The UCA1 level in 40 paired gastric adenocarcinoma and corresponding adjacent non-tumorous gastric tissue was detected by qPCR. Results were exhibited according to Lauren classification and analyzed by One-way ANOVA. **p < 0.01. **c** Representative images of in situ hybridization showing UCA1 expression within GC and control tissues. The relative density of UCA1 signal was quantified by Image J, and the significant was determined by student’s t-test. Scale bar, 100 μM. **d** The survival data containing 320 intestinal GC, 241 diffused GC and 32 mixed GC were analyzed by Kaplan-Meier analysis and *p* < 0.05 was considered statistically significant

UCA1 is a lncRNA whose expression is found to related to the initiation and progression of many kinds of cancers. To explore its function in GC, we first analyzed the expression of UCA1 in GC tissues and noncancerous control tissues by using two published microarray datasets, GSE54129 (111 GC patients and 21 control) and GSE65801 (32 paired GC and noncancerous tissues). We found a significantly overexpressed UCA1 in patients with GC (Fig. 1a). We also collected 40 paired GC and noncancerous tissue samples (21 intestinal, 13 diffused and 7 mixed) and examined the UCA1 level by qRT-PCR. We found UCA1 level only up-regulated in the intestinal GC subtype tissues significantly (Fig. 1b). To further confirm up-regulation of UCA1 in GC tissues and identify its subcellular location, we detected the UCA1 in GC and control tissue sections using in situ hybridization (ISH). As shown in Fig. 1c, dense UCA1 signal only was found in GC tissues and mainly existed in the cytoplasm. After quantification and statistical analysis, the ISH results indicated that UCA1 was overexpressed in GC tissues. Finally, we analyzed the data reported by Szász AM et al. (320 intestinal GC, 241 diffused GC and 32 mixed), and found intestinal GC patients with higher UCA1 level have a poor overall survival, suggesting UCA1 has a potential oncogenic role during the carcinogenesis of GC(Fig. 1d) [18].

### UCA1 functioned as an oncogene promoted proliferation, migration and inhibit apoptosis in GC cells

**Fig. 2:**
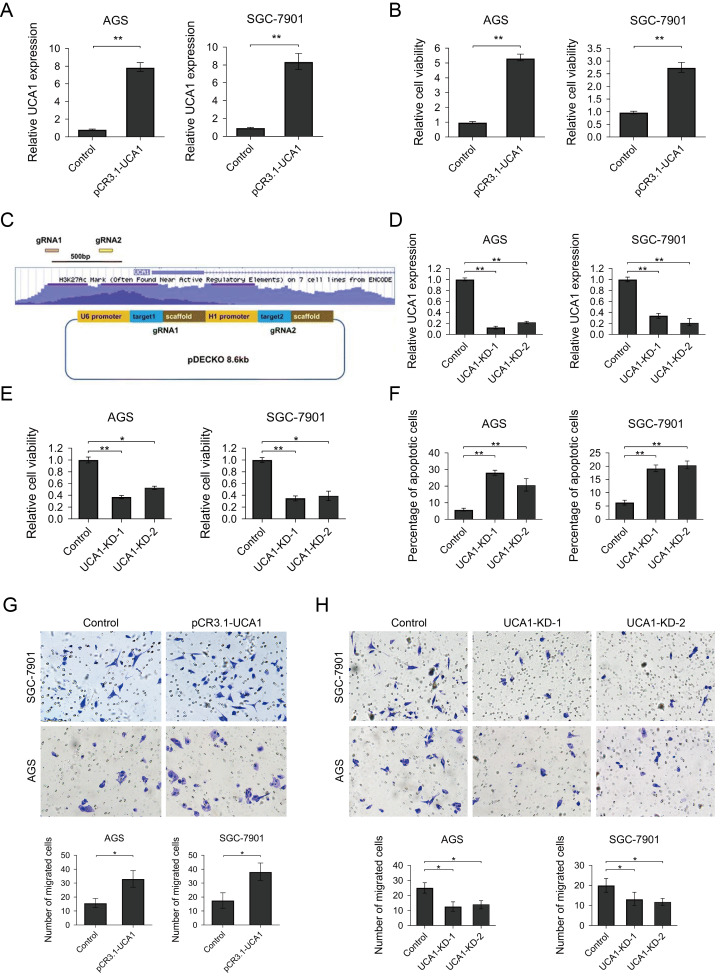
UCA1 functions as an onco-lncRNA promotes GC cells proliferation, migration, and inhibits apoptosis. **a** UCA1 overexpression GC cells were successfully established. **b** MTT assay was used to determine the cell viability of UCA1 overexpression and control GC cells. **c** Schematic diagram indicates the UCA1 knock-out vector design. Two guide RNAs targeting the promoter region of UCA1 were co-expressed by one plasmid. **d** UCA1 level was successfully reduced by co-transfecting UCA1-KD vector and Cas9 expression vector in two GC cells. **e** MTT assay to determine the cell viability of UCA1-KD GC cells. **f** Apoptosis assay. UCA1-KD or control GC cells were incubated with FITC labeled Annexin V antibody and then stained by PI. The percentage of apoptosis cells were determined by flow cytometry. **g** and **h** cells were deprived of serum overnight, treated with mitomycin-C and introduced into the upper chamber of the Transwell. Cells that migrated to the lower chambers were fixed with 4% paraformaldehyde and then stained with crystal violet. Crystal violet-stained cells were counted in 5 randomly different fields with an inverted microscope. Results were analyzed by student’s t-test and *p* < 0.05 was considered statistically significant. *p < 0.05, **p < 0.01

To further explore the biological function of UCA1 in the GC cells, we first constructed UCA1 overexpression vector and established UCA1 overexpression GC cell lines (Fig. 2a). As shown in Fig. 2b, GC cells with overexpressed UCA1 have significantly increased cell viability. Meanwhile, we constructed UCA1 knockdown vector which expresses two guide RNAs targeting the promoter region of UCA1 and then established UCA1 knockdown cell lines (Fig. 2c, d). The UCA1 promoter region knockout was confirmed by genotyping and sequencing(Additional file 1: Figure S1). As shown in Additional file 1: Figure S1A, The wildtype PCR product is 1705 bp which between bands of 1500 bp and 2000 bp of the marker lane. Meanwhile, the expected UCA1 knockdown PCR products are around 1103 bp which located between bands of 1000 bp and 1500 bp of the marker lane.

We found significantly reduced viability and increased apoptosis in the UCA1 knockdown cells (Fig. 2e, f). Subsequently, examined by transwell assay, UCA1 overexpression GC cells exhibited increased motility and UCA1 knockout GC cells have reduced migration cell numbers (Fig. 2g, h).

### UCA1 interacted with miR-26a/b and regulated the expression of EZH2 and CDK6

**Fig. 3:**
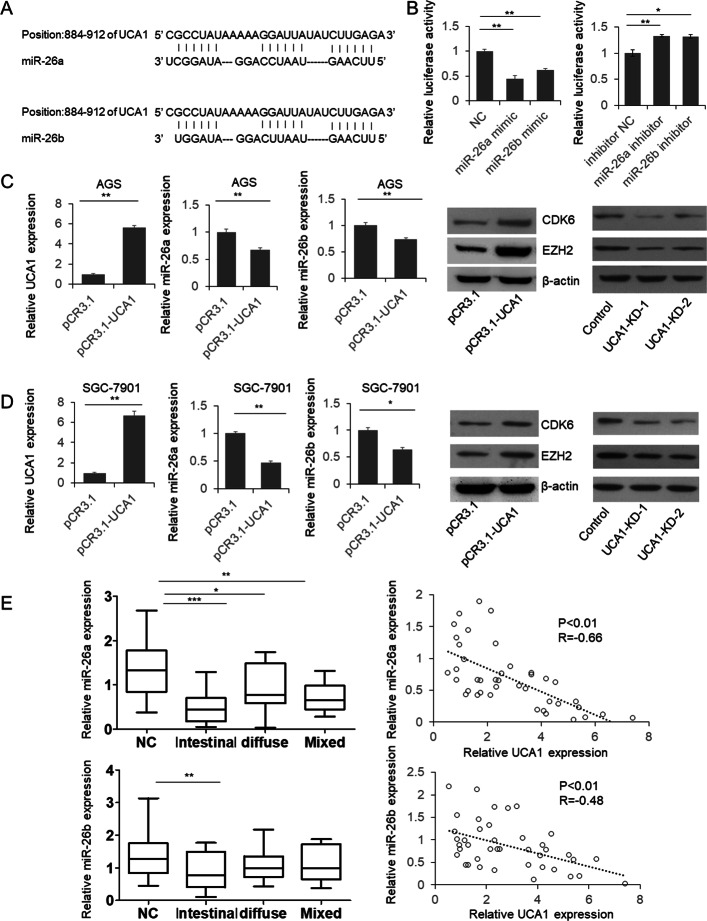
UCA1 sponges miR-26a/b and promote the expression of miR-26a/b target genes. **a** predicted interaction between miR-26a/b and UCA1. **b** dual luciferase assay. HEK293T cells transfected with UCA1 3’UTR reporter vector and miR-26a/b mimic or inhibitor. Relative luciferase activity was detected 48 h post interaction. **c** and **d** miR-26a and -b levels were repressed in UCA1 overexpression GC cells, and two miR-26a and -b target genes are up-regulated. **e** the level of miR-26a and -b in 40 paired GC and adjacent non-tumor control samples was determined by qRT-PCR. The Correlation between UCA1 and miR-26a or -b was analyze by correlation analysis

Accumulating evidence indicates that lncRNAs can exert “sponge-like” effects on various miRNAs, which subsequently inhibits miRNA-mediated functions [19]. So, we firstly analyzed the potential of interaction between UCA1 and miRNAs. We found miR-26a and -b may bind with UCA1 directly, and this binding was subsequently confirmed by dual-luciferase assay (Fig. 3a, b), RAP assay (Additional file 3: Figure S3A) and FISH (Additional file 3: Figure S3B). Meanwhile, when UCA1 was overexpressed in the GC cells, the expression of endogenous miR-26a and -b was significantly downregulated Fig. 3c and d. We also detected the protein level of two miR-26 targets EZH2 and CDK6 that were confirmed by other researchers [20, 21]. As shown in the right panel of Fig. 3c and d, the level of EZH2 and CDK6 was significantly increased in the UCA1 overexpression cells, suggesting the function of miR-26 was repressed. To further determine miR-26a and -b level in vivo, we detected miR-26a and -b expression using qRT-PCR. We found decreased miR-26a in all the GC tissues and decreased miR-26b in intestinal GC tissues (Fig. 3e). We also found a significant strong negative correlation between UCA1 and miR-26b (Fig. 3e).

To verify that UCA1 regulate EZH2 and CDK6 expression through sponging miR-26a and miR-26b, we first quantified the EZH2 and CDK6 protein level in tumour and control tissues by immunoblotting. Results indicated that both EZH2 and CDK6 protein were increased in GC tissues (Additional file 2: Figure S2A) and negatively correlated with UCA1 level(Additional file 2: Figure S2B and C). Meanwhile, in the UCA1 siRNA treated cells, EZH2 and CDK6 expressions were repressed, which can be rescued by miR-26a/b antagonists(Additional file 2: Figure S2D). These results indicated that UCA1 regulated EZH2 and CDK6 expression through sponging miR-26a/b.

### UCA1 directly interact with miR-214 and miR-193a

**Fig. 4:**
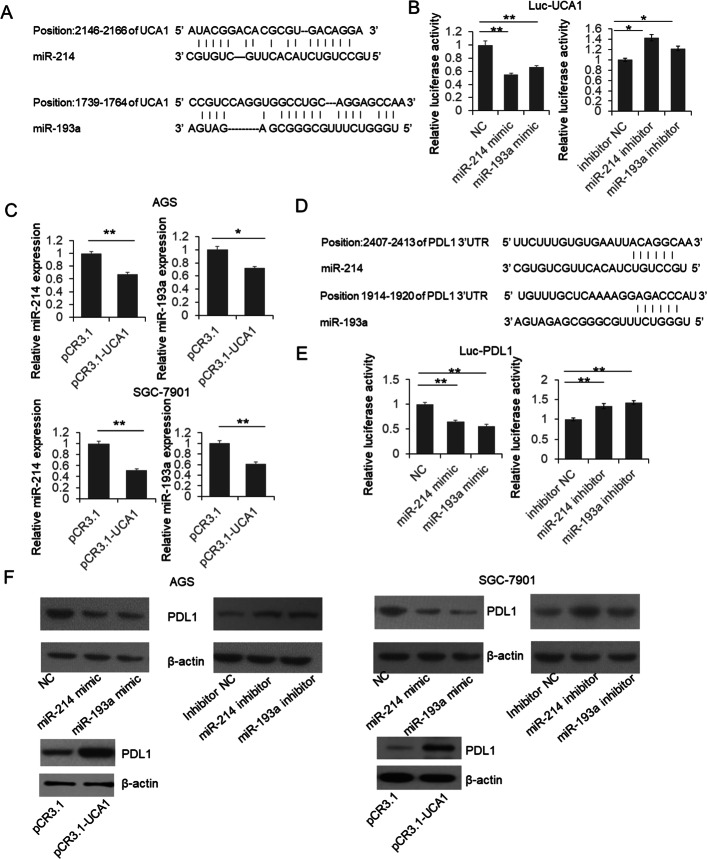
UCA1 interacts with miR-193a and miR-214, and then modulate PDL1 expression. **a** predicted interaction between UCA1 and miR-193a or miR-214. **b** dual luciferase assay. HEK293T cells were co-transfected with reporter vector using full sequence of UCA1 as 3’UTR of firefly luciferase, and miRNAs mimics or inhibitors. The luciferase activity was detected 48 h post transfection. **c** The relative miR-193a and − 214 expression was determined by qRT-PCR. **d** predicted interaction between PDL1 and miR-193a or miR-214. **e** dual luciferase assay. HEK293T cells were co-transfected with PDL1 3’UTR reporter vector and the mimic or inhibitor of miR-193a or miR-214 for 48 h. Luciferase activities were detected and results were analyzed by student’s t-test. **f** miR-193a and miR-214 repress endogenous PDL1 expression

In addition, we also found miR-214 and -193 have the potential to be ‘sponged’ by UCA1 (Fig. 4a), and both were reported function as tumor suppressors [22, 23]. Dual luciferase assay was processed to confirm the interaction between miRNAs and UCA1. As shown in Fig. 4b, luciferase activities were repressed by miR-193a and − 214 mimics, and up-regulated by miR-193a and − 214 inhibitors, meanwhile, the level of miR-193a and − 214 was reduced in UCA1 overexpression cells (Fig. 4c). In the GC tissues, both of miR-193 and miR-214 were reduced and negatively correlated with UCA1 level (Additional file 4: Figure S4A), indicating the direct interaction between miRNAs and UCA1. The direct interaction between UCA1 and miR-193 or miR-214 was confirmed by RAP assay (Additional file 3: Figure S3A) and FISH (Additional file 3: Figure S3B) in GC cells.

### UCA1 regulated PDL1 expression through sponging miR-214 and miR-193a

PDL1 is an important member of the B7 family molecules which can interact with PD-1 to inhibit the T-cell activation, induce apoptosis of effector T cells, and finally impair the anti-tumor immunity [24, 25]. Increasing evidences indicated that PDL1 overexpression in many human cancers is significantly associated with tumor progression and patient’s prognosis [26, 27]. Predicted by online bioinformatics tools, we surprisingly found miR-214 and -193a target sites in the 3’UTR of PDL1 Mrna (Fig. 4d). Subsequent dual-luciferase assay indicated that miR-214 and -193a can repress luciferase activity by targeting the 3’UTR of PDL1(Fig. 4e). Meanwhile, in the gastric cancer cells, the endogenous PD-L1 expression was also modulated by miR-214 and -193a (Fig. 4f). In intestinal GC tissues, PD-L1 expression was found increased and positively correlated with UCA1 level(Additional file 4: Figure S4B).

**Fig. 5:**
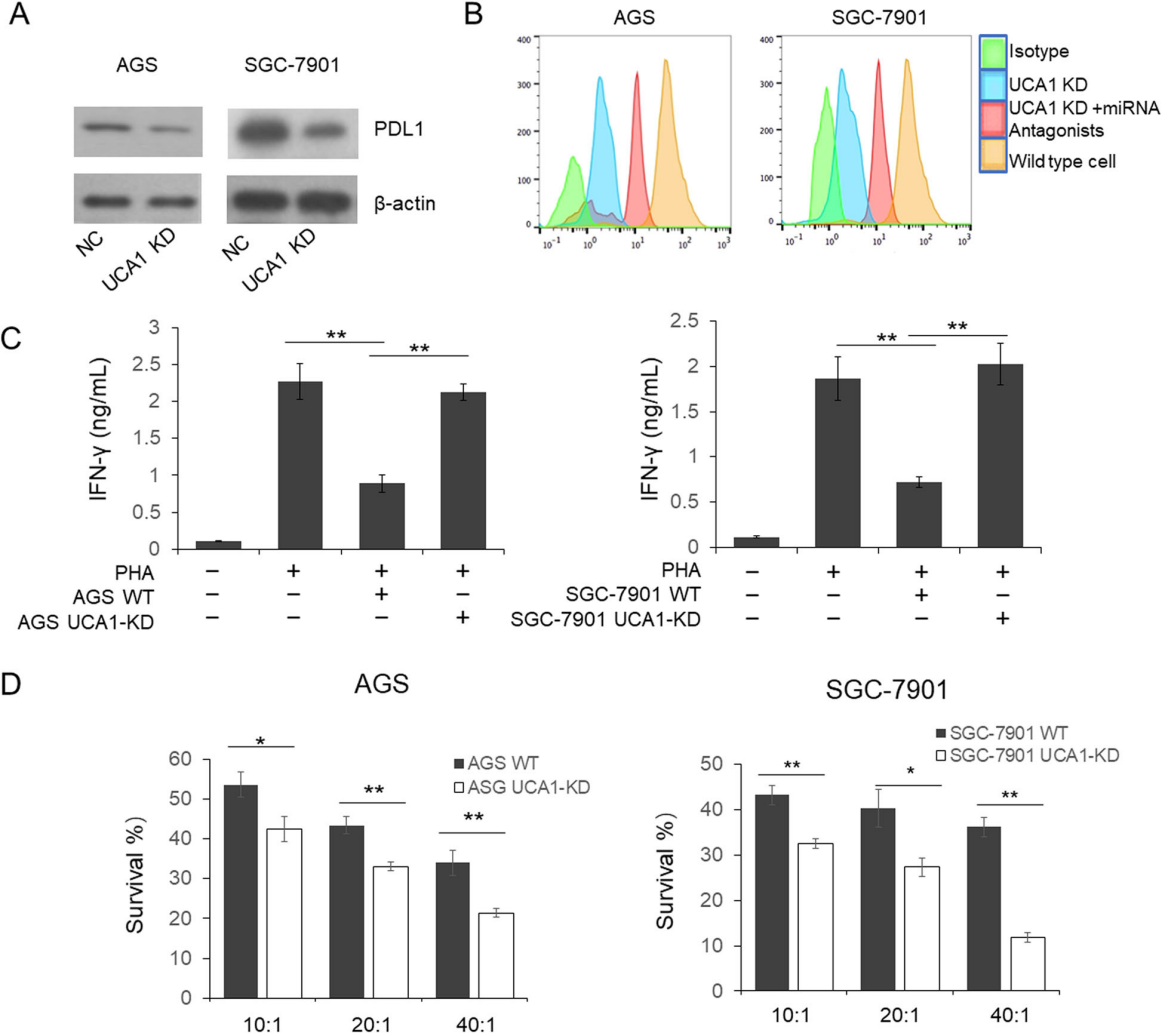
knockout of UCA1 restored PHA-stimulated T cell activation and improved the cytotoxicity of CIK therapy. **a** total PDL1 level was reduced in UCA1-KD GC cells. **b** cell surface PDL1 was detected by flow cytometry. **c** PHA induced PBMCs were co-cultured with UCA1-KD or control GC cells. IFNγ production in the supernatant was detected by ELISA. **d** knockdown of PDL1 expression in GC cells could improve the cytotoxic sensitivity to CIK therapy in vitro

To further determine whether UCA1 knock out can induce PDL1 downregulation in GC cells by sponging miRNAs, and subsequently alleviate T cell suppression, we first analyzed the PDL1 in the UCA1 knock out GC cells by western blot and flow cytometry. We found a significant reduced PDL1 expression and left shift peak of the UCA1-KO GC cells (Fig. 5a,b). Meanwhile, the peak left shift can partially rescued by antagonists for miR-193a and miR-214 suggesting the cell surface PDL1 level was regulated by UCA1-miR-193a/214 axis.

### UCA1 regulated PDL1 expression and modulate anti-tumour immune response

Subsequently, we evaluated if modulation of PDL1 expression on GC cell lines could alter PHA-stimulated activation of healthy donor PBMC as measured by the released supernatant IFNγ level. As shown in Fig. 5c, IFNγ release by PHA stimulated PBMC was partially suppressed by the AGS-NC or SGC7901-NC cells when compared with PHA-stimulated PBMC alone suggesting a significant PDL1 mediated immune suppressive effect. When PBMCs co-cultured with UCA-KO GC cells, the IFNγ levels were increased, indicated that PDL1 downregulation induced by UCA1 knock out alleviated the immune suppressive effect. Subsequently, we analyzed the cytotoxicity of CIK cells on both AGS and SGC-7901 cells when the effector-target ratio was in the range 10:1 to 40:1. As shown in Fig. 5d, the cancer cell survival rate was obviously related to the effector-target ratio, and decreased with increase of the effector-target ratio. The cytotoxic activity of the CIK cells against AGS-UCA1-KO cells was markedly higher than that of AGS-NC cells under the same conditions (Fig. 5d, *p* < 0.05), and the same in SGC-7901 cells.

**Fig. 6:**
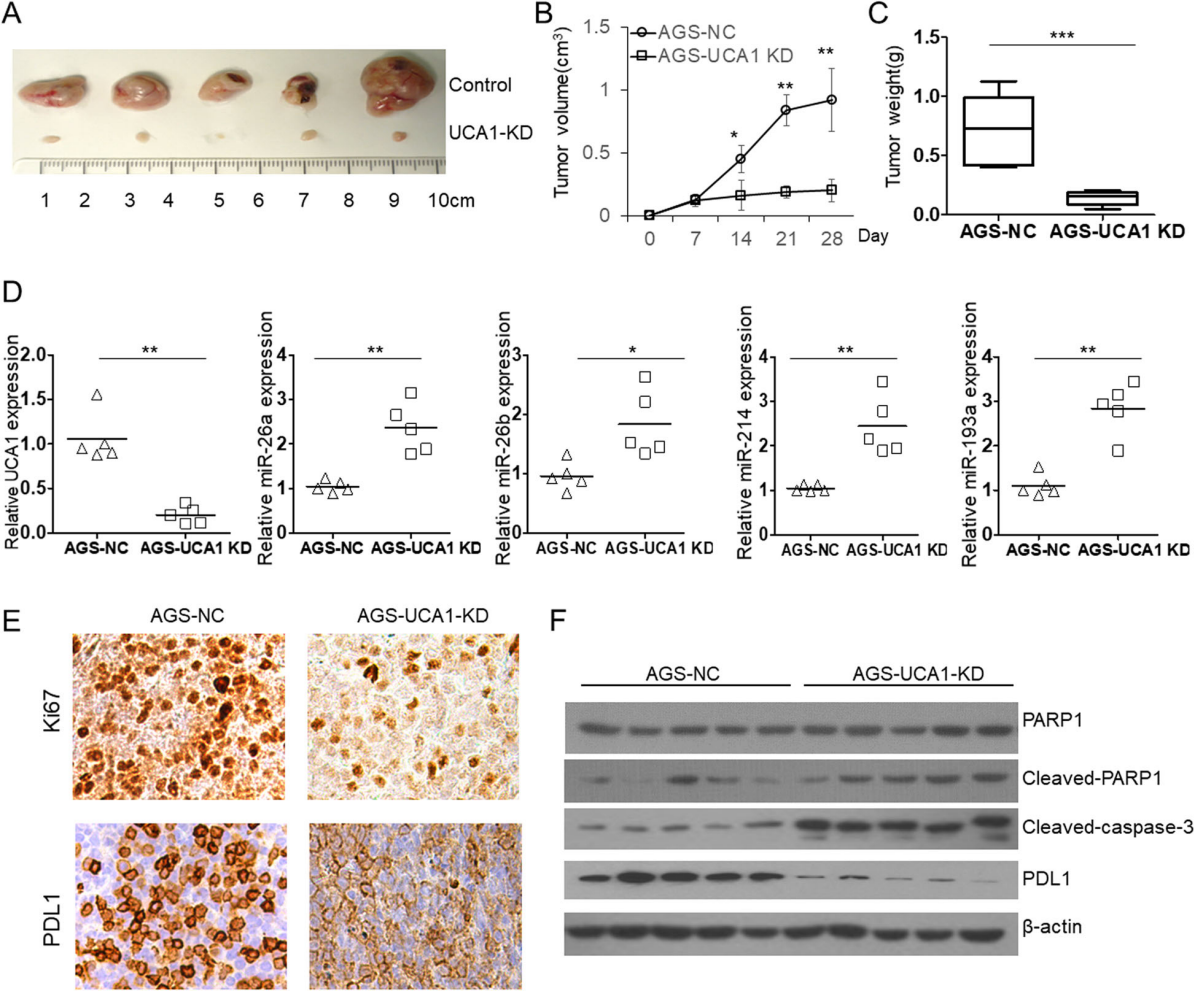
knockdown of UCA1 expression inhibit the tumor growth in xenograft mouse model. **a** we examined the effect of knockdown of UCA1 on the regulation of subcutaneous transplantation mouse model of gastric cancer by using AGS cell line. The AGS-NC and AGS-UCA1-KD cells were injected subcutaneously into SCID mice. The tumor volumes were measured every week for 4 weeks. **b** the statistical analysis showed that knockdown of PDL1 expression significantly inhibits tumor growth. **c** after 28 days, the tumor weight of UCA1-KD group was lighter than that of control group (*p* < 0.001). **d** the qRT-PCR results showed that the level of miR-26a, miR-26b, miR-193a and miR-214 was increased in the UCA1-KD group. **e** the immunohistochemistry results showed that PDL1 protein level as well as Ki-67 was down regulated in UCA1-KD group. **f** The immunoblotting results showed that cleaved PARP1 and caspase-3 level was increased in the UCA1-KD tumors

In order to explore the effect of UCA1 knockout in the regulation of tumor growth in vivo, we further examined the function of UCA1 using AGS cell subcutaneous xenograft mouse model. The AGS-NC and AGS-UCA1-KO cells were injected subcutaneously into SCID mice. The tumor volumes were measured every 7 days for 28 days. As shown in Fig. 6a, knockout of UCA1 significantly inhibit tumor growth in mice. After 30 days, the tumor volume and weight of AGS-UCA1-KO group were significant smaller and lighter than those of AGS-NC group (Fig. 6b, c). Meanwhile, the level of anti-tumor miRNAs (miR-26a, miR-26b, miR-193a and miR-214) that directly interact with UCA1, was increased in the UCA1-KO tumors (Fig. 6d). Decreased Ki-67, PDL1 and increased cleaved PARP1 and caspase-3 were also found in the UCA1-KO tumors (Fig. 6e and f).

## Discussion

LncRNAs have been identified to function as key regulators of diverse cellular processes including the initiation and progression of cancer. As an lncRNA, although UCA1 was found overexpressed in gastric cancer, the role of UCA1 in GC is still not fully understood. In this study, we first analyzed two newly released microarray datasets, and found the consistent results showing overexpressed UCA1 in the GC tissues. According to Lauren’s criteria, gastric cancer is classified into two major histological subtypes: intestinal type and diffuse type adenocarcinoma [28]. After analyzing the UCA1 level in 40 paired GC and adjacent non-tumor tissue samples, we found that UCA1 especially overexpressed in intestinal GC tissues. Since the two histological subtypes of gastric cancer have lots of distinct clinical and molecular characteristics, further analysis combine the UCA1 expression and other factors including molecular characteristics, drug sensitivity and biological behaviors, should be helpful to guide the GC clinical treatment.

MiR-26a and miR-26b are the two multiple functional miRNAs which were found dysregulated in diverse cancers and are involved in various biological processes, including proliferation, migration, invasion, angiogenesis, and metabolism by modulating different signaling pathways. MiR-26a level has been found to be reduced in the plasma and cancer tissue samples of GC patients, but the mechanism are still not known [29, 30]. We identified that UCA1 functions as a miRNA “sponge” modulating miR-26a and miR-26b level by direct interaction. Furthermore, we also identified the negative correlation between UCA1 level and miR-26a or miR-26b in GC tissue samples, which may provide an answer of how UCA1 modulate the level of miR-26a or miR-26b in GC.

PDL1 is a transmembrane protein that usually overexpressed in cancer cells, and plays a major role in suppressing the immune system by binding with PD1, which was found on activated T cells, B cells, and myeloid cells [31]. In gastric cancer, increasing evidences indicates that upregulated PDL1 level associates cancer progression and worse prognosis [32, 33]. In this study, we identified that miR-193a and − 214 repressed PDL1 expression by targeting 3’UTR. Meanwhile, we confirmed that UCA1 functioned as a “miRNA sponge” absorbs miR-193a and miR-214 and protect the PDL1 expression at RNA level. These results indicated that UCA1 targeting treatment may not only a treatment targeting the tumor cells, but also a therapy activates the host immune system. Furthermore, our research indicated that UCA1-KO GC cells could induced a higher IFNγ expression when co-cultured with PBMCs, and have a lower survival rate when co-cultured with CIK cells in vitro, suggesting UCA1-KO GC cells may be more easily to be eliminated by the immune system.

In conclusion, we present here that UCA1 functions as an onco-lncRNA promotes GC cells proliferation, migration and inhibits GC cells apoptosis by repressing anti-tumor miRNAs as miR-26a and miR-26a. Meanwhile, UCA1 also represses the host immune systems by up-regulating the PDL1 level of GC cells. UCA1-KO GC cells formed smaller tumors, and had higher miR-26a, −26b, −193a and − 214 level, reduced cell proliferation and increased apoptosis in vivo, indicating that UCA1 could serve as a potential novel therapeutic target for GC treatment.

## Additional files

Additional file 1: **Figure S1.** Establish UCA1 knockdown cells. AGS or SGC-7901 cells were co-transfected with pUCA1-KD and plentiCas9-Blast followed by selection for more than 5 days with blasticidin (4μg/ml) and puromycin (2μg/ml) for 1 week. The knockout of UCA1 promoter region was confirmed by genotyping and sequencing. (A) Genotyping results. The wildtype PCR product is 1705 bp and the expected bands are around 1103 bp. The lanes near the markers are wildtype PCR products. Two successful UCA1 knockdown clones in each cell line were labeled. Non-labeled lanes were the clones not used for functional study. (B)Sequencing results for UCA1 knockdown AGS clones. (C) Sequencing results for UCA1 knockdown SGC-7901 clones. (TIF 7729 kb)

Additional file 2: **Figure S2.** UCA1 regulate EZH2 and CDK6 expression dependent on sponging miR-26a/b. (A) immunoblotting quantify EZH2 and CDK6 protein level in tumors and control tissues from patients with intestinal GC. (B) correlation analysis between UCA1 and CDK6 level in tumors. (C) correlation analysis between UCA1 and EZH2 level in tumors. (D) miR-26a/b antagonists rescued the repressive function of UCA1 siRNAs on EZH2 and CDK6 expression. (TIF 3313 kb)

Additional file 3: **Figure S3.** UCA1 interact with miR-26a, miR-26b, miR-193a and miR-214 in GC cells and regulate their targets expression. (A) Cell extracts were incubated with biotin labelled UCA1 probe, with biotin labelled sequence scrambled DNA oligo as control. Hybridized material was captured with magnetic streptavidin beads. Beads were subjected to RNA extraction followed by RT-qPCR to quantify miRNAs levels. (B) Fluorescence in situ hybridization to identify the colocalization between UCA1 and miRNAs. (C) miRNAs expressions were quantified by RT-qPCR in UCA1 knockdown cells. (D) The protein level of TAK1(miR-26a/b target), KRAS(miR-193a target) and FGF9 was examined by immunoblotting in UCA1 overexpression or knockdown GC cells (TIF 14 kb)

Additional file 4: **Figure S4.** miR-193a and miR-214 regulated PDL1 expression and modulated immune response. (A) miR-193a and miR-214 levels were examined by RT-qPCR in tumor and control tissues from patients with GC. The correlation between UCA1 and miRNAs were analyzed. (B) PDL1 protein level was quantified by immunoblotting in tumor and control tissues from patients with GC. The correlation between PDL1 and UCA1 was analyzed. (C) PHA induced PBMCs were co-cultured with miRNA mimic transfected or control GC cells. IFNγ production in the supernatant was detected by ELISA. (D) miR-193a and miR-214 mimics transfection improve the cytotoxic sensitivity to CIK therapy in vitro. (TIF 6730 kb)

## Data Availability

The datasets used and/or analyzed during the current study are available from the corresponding author on reasonable request.

## Abbreviations

ceRNA: endogenous RNA
CIK: Cytokine-induced killer
FBS: Fetal bovine serum
GC: Gastric cancer
KO: Knock out
lncRNAs: Long non-coding RNAs
miRNAs: Micro RNAs
OD: Optical density
PBMCs: Peripheral blood mononuclear cells
SCID: Severe combined immunodeficient
UTR: Untranslated region
WT: Wild type
