# Correction to: The lncRNA UCA1 promotes proliferation, migration, immune escape and inhibits apoptosis in gastric cancer by sponging anti-tumor miRNAs

**DOI:** 10.1186/s12943-019-1059-2

**Published:** 2019-08-27

**Authors:** Chao-Jie Wang, Chun-Chao Zhu, Jia Xu, Ming Wang, Wen-Yi Zhao, Qiang Liu, Gang Zhao, Zi-Zhen Zhang

**Affiliations:** Department of Gastrointestinal Surgery, Ren Ji Hospital, School of Medicine, Shanghai Jiao Tong University, No. 160 Pu Jian Road, Shanghai, 200127 China

**Correction to: Mol Cancer**

**https://doi.org/10.1186/s12943-019-1032-0**

Following publication of the original article [1], authors reported Pro. Gang Zhao has to be considered as another corresponding author, according to his important contribution. In the original submission, Pro. Gang Zhao has been already listed as a joint corresponding author. But there was a mistake about author list in the proof process. About the formal author list, please see above author group section.
