# Correction to: The lncRNA UCA1 promotes proliferation, migration, immune escape and inhibits apoptosis in gastric cancer by sponging anti-tumor miRNAs

**DOI:** 10.1186/s12943-021-01387-7

**Published:** 2021-09-18

**Authors:** Chao-Jie Wang, Chun-Chao Zhu, Jia Xu, Ming Wang, Wen-Yi Zhao, Qiang Liu, Gang Zhao, Zi-Zhen Zhang

**Affiliations:** Department of Gastrointestinal Surgery, Ren Ji Hospital, School of Medicine, Shanghai Jiao Tong University, No. 160 Pu Jian Road, Shanghai, 200127 China

**Correction to: Mol Cancer 18, 115 (2019)**

**https://doi.org/10.1186/s12943-019-1032-0**

Following publication of the original article [1], the authors identified some minor errors in image-typesetting in Fig. 2; specifically in Fig. 2g and h (all panels corrected). The corrected figure is given here. The correction does not have any effect on the results or conclusions of the paper.

**Fig. 2:**
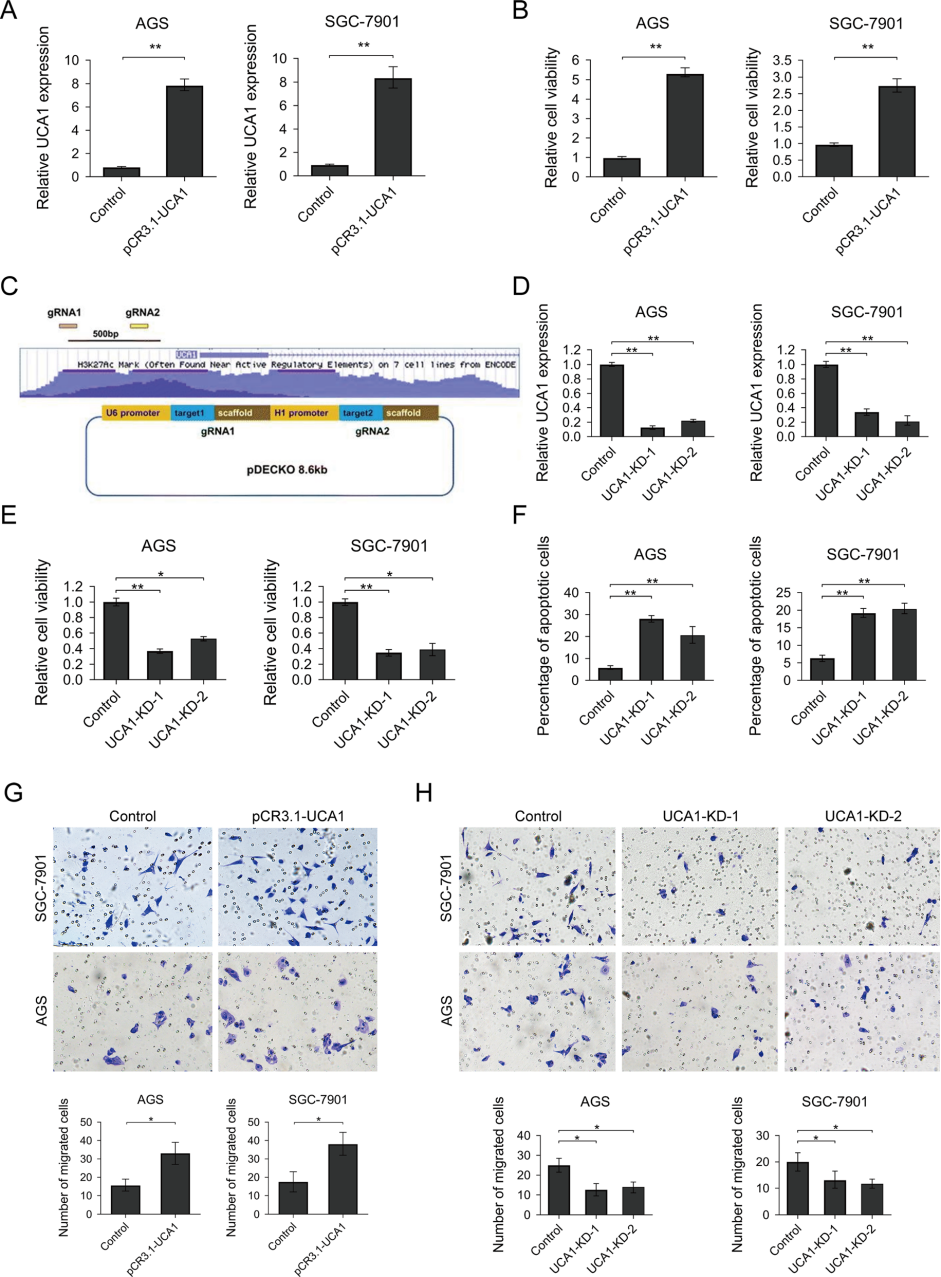
UCA1 functions as an onco-lncRNA promotes GC cells proliferation, migration, and inhibits apoptosis. **a** UCA1 overexpression GC cells were successfully established. **b** MTT assay was used to determine the cell viability of UCA1 overexpression and control GC cells. **c** Schematic diagram indicates the UCA1 knock-out vector design. Two guide RNAs targeting the promoter region of UCA1 were co-expressed by one plasmid. **d** UCA1 level was successfully reduced by co-transfecting UCA1-KD vector and Cas9 expression vector in two GC cells. **e** MTT assay to determine the cell viability of UCA1-KD GC cells. **f** Apoptosis assay. UCA1-KD or control GC cells were incubated with FITC labeled Annexin V antibody and then stained by PI. The percentage of apoptosis cells were determined by flow cytometry. **g** and **h** cells were deprived of serum overnight, treated with mitomycin-C and introduced into the upper chamber of the Transwell. Cells that migrated to the lower chambers were fixed with 4% paraformaldehyde and then stained with crystal violet. Crystal violet-stained cells were counted in 5 randomly different fields with an inverted microscope. Results were analyzed by student’s t-test and *p* < 0.05 was considered statistically significant. *p < 0.05, **p < 0.01

The original article has been updated.
