# Supplementary figures and images for: The lncRNA UCA1 promotes proliferation, migration, immune escape and inhibits apoptosis in gastric cancer by sponging anti-tumor miRNAs
Source: Mol Cancer. 2019 Jul 4;18:115. doi: 10.1186/s12943-019-1032-0 (PMC6609402; doi:10.1186/s12943-019-1032-0)

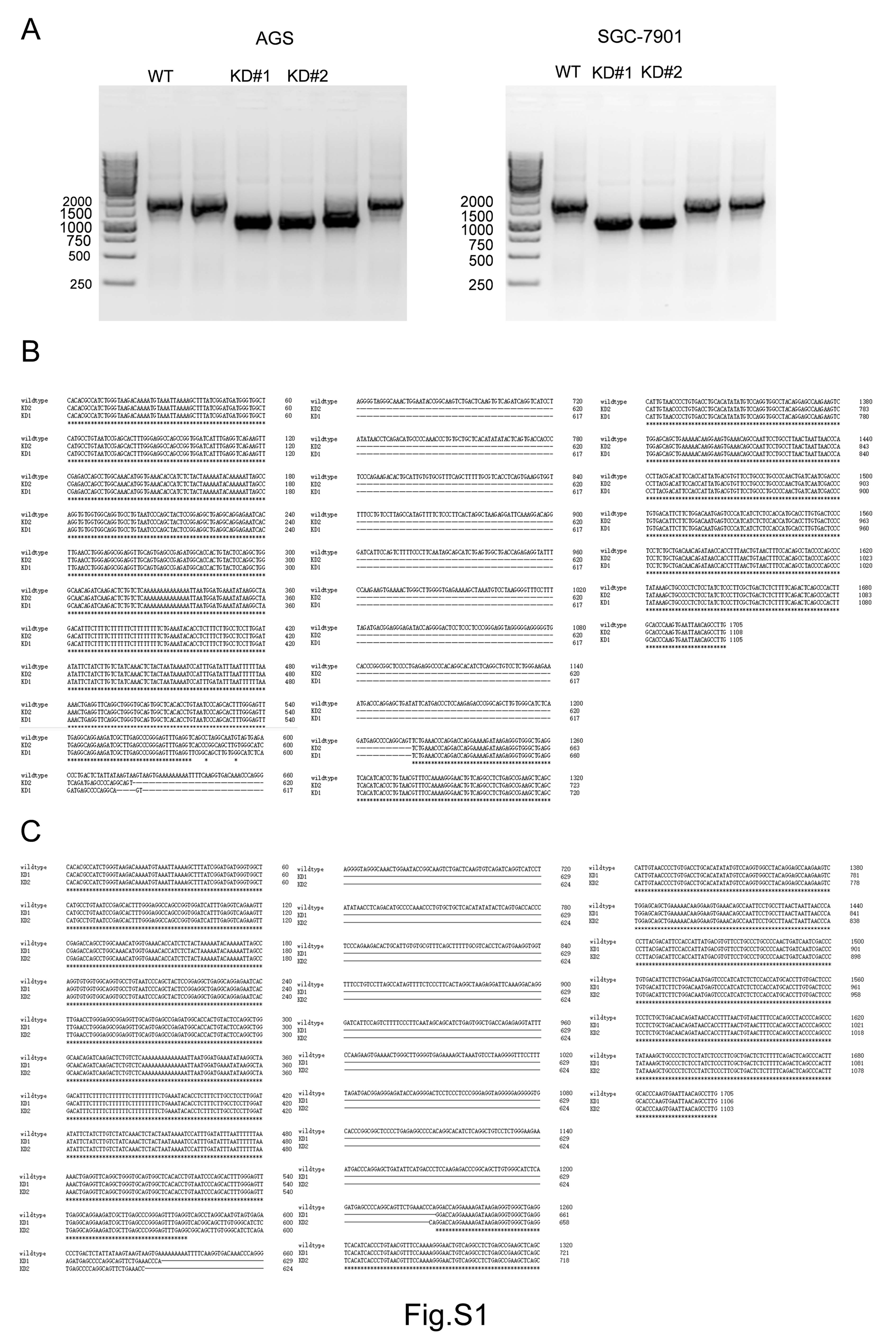

Supplement: Supplementary file 1 — Figure S1. Establish UCA1 knockdown cells. AGS or SGC-7901 cells were co-transfected with pUCA1-KD and plentiCas9-Blast followed by selection for more than 5 days with blasticidin (4μg/ml) and puromycin (2μg/ml) for 1 week. The knockout of UCA1 promoter region was confirmed by genotyping and sequencing. (A) Genotyping results. The wildtype PCR product is 1705 bp and the expected bands are around 1103 bp. The lanes near the markers are wildtype PCR products. Two successful UCA1 knockdown clones in each cell line were labeled. Non-labeled lanes were the clones not used for functional study. (B)Sequencing results for UCA1 knockdown AGS clones. (C) Sequencing results for UCA1 knockdown SGC-7901 clones. (TIF 7729 kb) [file 12943_2019_1032_MOESM1_ESM.tif]

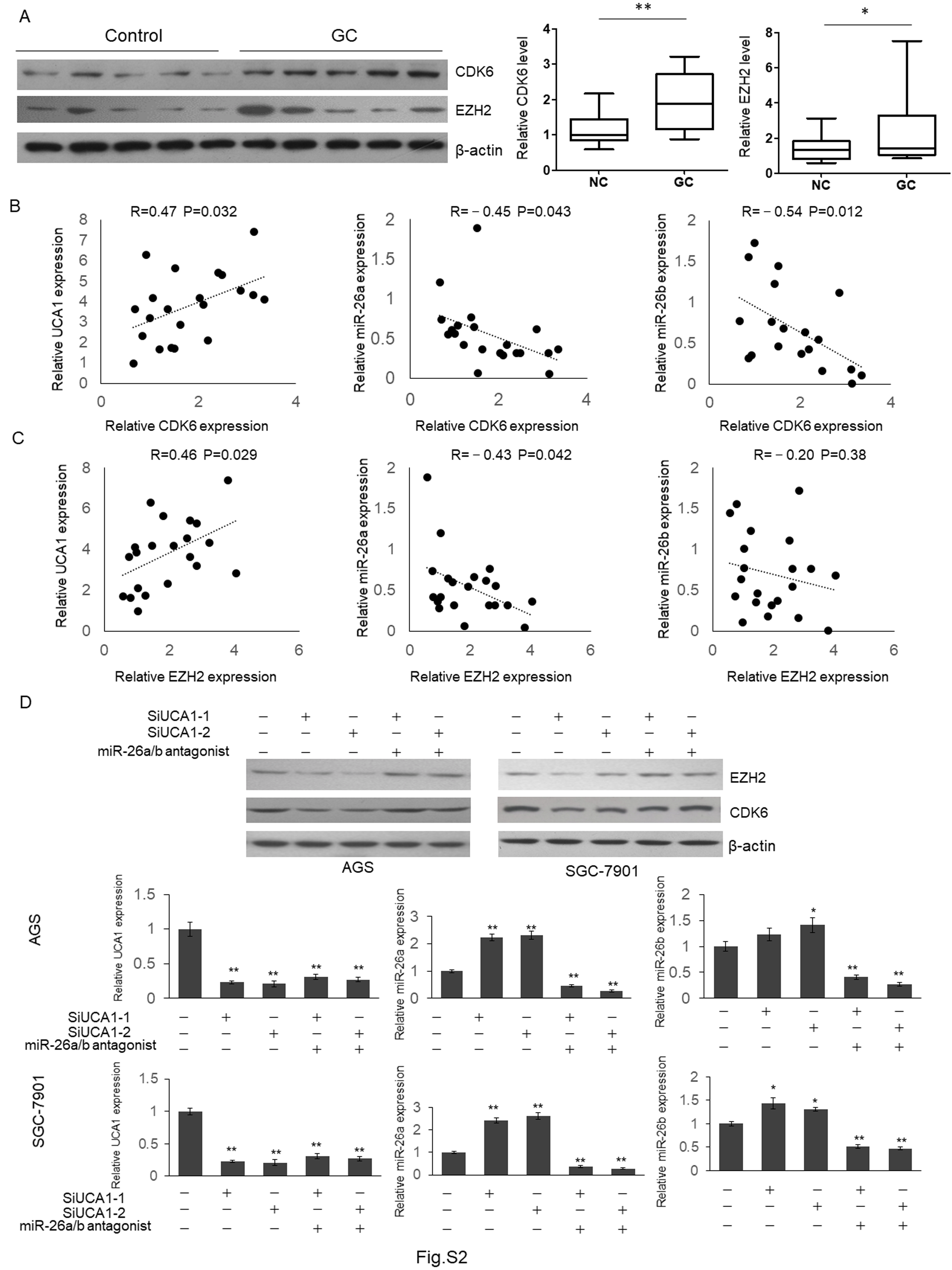

Supplement: Supplementary file 2 — Figure S2. UCA1 regulate EZH2 and CDK6 expression dependent on sponging miR-26a/b. (A) immunoblotting quantify EZH2 and CDK6 protein level in tumors and control tissues from patients with intestinal GC. (B) correlation analysis between UCA1 and CDK6 level in tumors. (C) correlation analysis between UCA1 and EZH2 level in tumors. (D) miR-26a/b antagonists rescued the repressive function of UCA1 siRNAs on EZH2 and CDK6 expression. (TIF 3313 kb) [file 12943_2019_1032_MOESM2_ESM.tif]

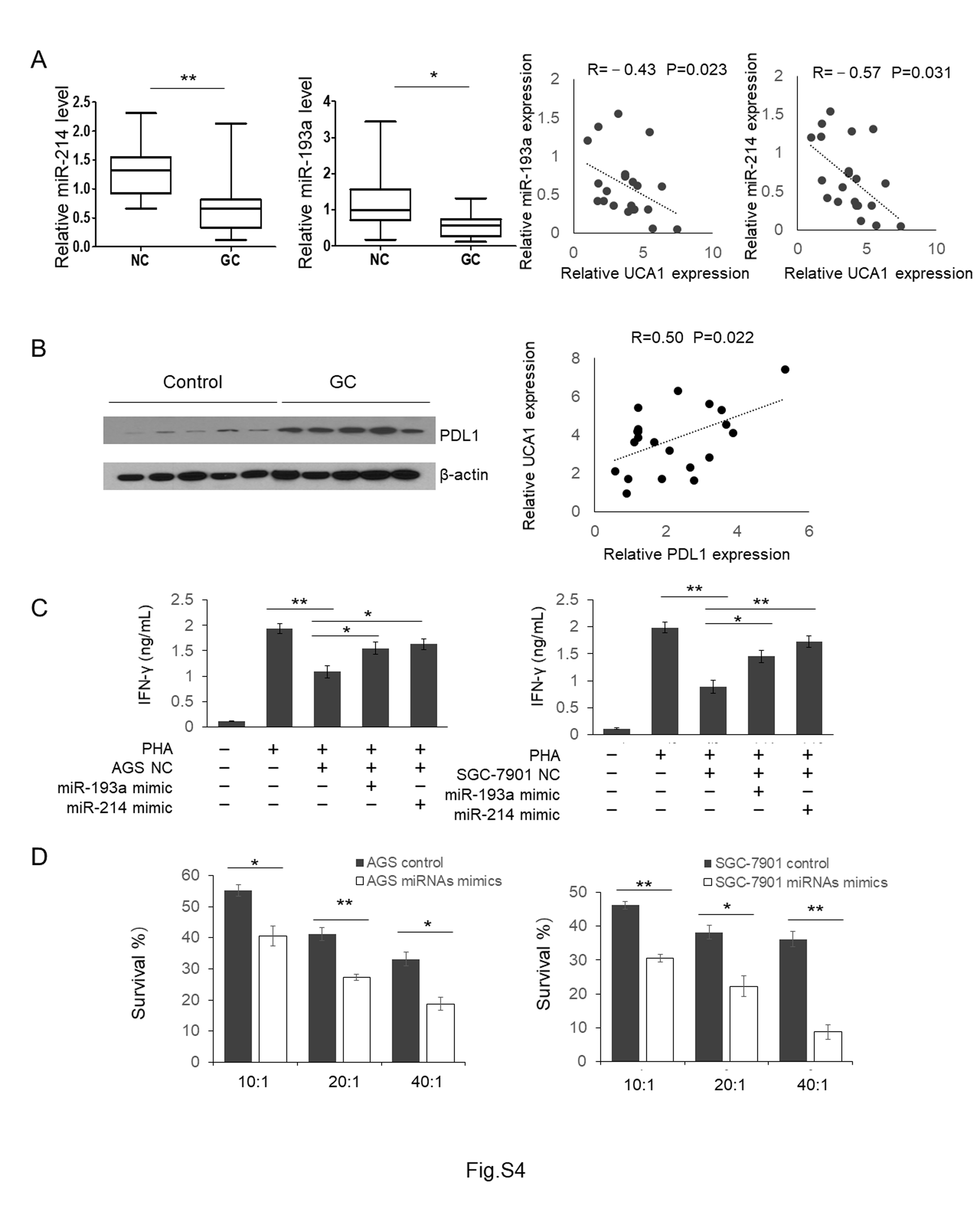

Supplement: Supplementary file 4 — Figure S4. miR-193a and miR-214 regulated PDL1 expression and modulated immune response. (A) miR-193a and miR-214 levels were examined by RT-qPCR in tumor and control tissues from patients with GC. The correlation between UCA1 and miRNAs were analyzed. (B) PDL1 protein level was quantified by immunoblotting in tumor and control tissues from patients with GC. The correlation between PDL1 and UCA1 was analyzed. (C) PHA induced PBMCs were co-cultured with miRNA mimic transfected or control GC cells. IFNγ production in the supernatant was detected by ELISA. (D) miR-193a and miR-214 mimics transfection improve the cytotoxic sensitivity to CIK therapy in vitro. (TIF 6730 kb) [file 12943_2019_1032_MOESM4_ESM.tif]
